# Implicit consequentiality bias in English: A corpus of 300+ verbs

**DOI:** 10.3758/s13428-020-01507-z

**Published:** 2020-12-02

**Authors:** Alan Garnham, Svenja Vorthmann, Karolina Kaplanova

**Affiliations:** School of Psychology, University of Sussex, Pevensey 1 Building, Falmer, Brighton, BN1 9QH UK

**Keywords:** Psycholinguistics, Verbs, Thematic roles, Consequentiality, Causality, Corpus studies

## Abstract

This study provides implicit verb consequentiality norms for a corpus of 305 English verbs, for which Ferstl et al. (*Behavior Research Methods, 43,* 124-135, 2011) previously provided implicit causality norms. An online sentence completion study was conducted, with data analyzed from 124 respondents who completed fragments such as “John liked Mary and so…”. The resulting bias scores are presented in an Appendix, with more detail in supplementary material in the University of Sussex Research Data Repository (via 10.25377/sussex.c.5082122), where we also present lexical and semantic verb features: frequency, semantic class and emotional valence of the verbs. We compare our results with those of our study of implicit causality and with the few published studies of implicit consequentiality. As in our previous study, we also considered effects of gender and verb valence, which requires stable norms for a large number of verbs. The corpus will facilitate future studies in a range of areas, including psycholinguistics and social psychology, particularly those requiring parallel sentence completion norms for both causality and consequentiality.

Language researchers have long used normative data both to investigate effects such as that of frequency on word identification and to control for those effects when other, more subtle, influences on those processes are under investigation. When large-scale norms were time-consuming to collect and score, only commonly used measures received systematic treatment, with word frequency being the paradigm example. For less commonly investigated features, for example implicit causality of verbs, small-scale norms were often collected for individual studies. More recently, norms have become easier to collect and score, and a number of factors have driven the need for norms on larger sets of items, in particular the use of techniques such as EEG and functional magnetic resonance imaging (fMRI) that require large sets of items if effects are to stand out from a background of noise, and the replication crisis, which suggests the use of larger sets of items (and participants) in all studies. For example, an event-related potential (ERP) study by Misersky, Majid, and Snijders (2019) used the large set of 400+ gender stereotype norms collected by Misersky et al. (2014), which have also been used in a range of other studies (e.g., Lewis & Lupyan, 2020; Richy & Burnett, 2020; Mueller-Feldmeth, Ahnefeld, & Hanulikova, 2019; Gygax et al., 2019). Studies of the effect of emotional valence on word recognition times (Citron, Weekes, & Ferstl, 2012) and on ERP components during word recognition (Citron, Weekes, & Ferstl, 2013) used the Sussex Affective Word List (SAWL) with ratings on 525 words, and a more recent study by Chen et al. (2015) used the alterative ANEW corpus (Affective Norms for English Words, Bradley & Lang, 1999), which has an even larger set of ratings, in this case for American English. Our own set of implicit causality norms (Ferstl, Garnham, & Manouilidou, 2011) has been used in a wide range of studies (e.g., Cheng & Almor, 2019; Van den Hoven & Ferstl, 2018; Dresang & Turkstra, 2018; Wang et al., 2017; Hartshorne, 2014). In addition, Hartshorne has published some re-analyses of our data, which only make sense because of the size of our corpus (Hartshorne & Snedeker, 2013; Hartshorne, Sudo, & Uruwashi, 2013). Measures of word frequency have also benefitted from modern techniques. For example, the SUBTLEX-UK norms for British English (Van Heuven, Mandera, Keuleers, & Brysbaert, 2014) are based on a corpus of around 200 million tokens, compared with the one million-word Brown Corpus that was used to create the classic Kučera and Francis (1967) norms, and have advantages over other sets of norms (see Van Heuven et al., 2014, for details). Another recent set of norms with multiple measures for a very large number of words (5000+) is the Glasgow norms (Scott, Keitel, Becirspahic, Yao, & Sereno, 2018).

The implicit causality norms of Ferstl et al. (2011) are based on a corpus of over 300 verbs. The norms were collected in an online study in which participants completed sentence fragments of the form “John liked Mary because…”. For each verb, the bias towards selecting one or other of the protagonists (denoted by the first and second names, referred to as NP1 and NP2) as the cause was calculated by looking at the number of completions that began with a reference to one of the NPs as a proportion of the number that began with a reference to one or the other (but not both or neither). The verbs denoted a mix of actions and states, both of which have causes, and understanding a narrative properly requires computation of the causal relations between the events and the states described in it (Graesser, Singer & Trabasso, 1994). The verbs were grouped into four classes, derived from previous literature, according to the thematic roles assigned to the NP1 and the NP2: Experiencer-Stimulus, Stimulus-Experiencer, Agent-Patient, and Agent-Evocator. Semantic analysis associates causation with Stimulus, Stimulus, Agent, and Evocator, respectively, in the four classes, and there is a debate about how this all-or-none classification of causes relates to the biases of various strengths that emerge in norming studies (e.g., Crinean & Garnham, 2006; Pickering & Majid, 2007).

If one event or state is the cause of another, the second is the effect or consequence of the first. And although a cause typically precedes its consequences, the same event will have both causes, which precede it, and consequences, which follow it. It is therefore not surprising that, in addition to the phenomenon of implicit causality, the phenomenon of implicit consequentiality has also been identified in the literature (Au, 1986; Stewart, Pickering, & Sanford, 1998a), and like implicit causality, implicit consequentiality affects language processing (e.g., Au, 1986; Stewart et al., 1998a; Rigalleau, Guerry, & Granjon, 2014), though it is not as well studied as implicit causality. Furthermore, an analysis based on thematic roles (Crinean & Garnham, 2006) suggests that for three of the four classes of verbs (Experiencer-Stimulus, Stimulus-Experiencer, Agent-Patient) the implicit consequence^1^ is the other NP than the implicit cause, but for Agent-Evocator verbs, it is the same, namely the Evocator. Crinean and Garnham showed that these relations held in a small corpus of implicit causality and consequentiality norms collected by Stewart, Pickering, and Sanford (1998b), but they have not been established more generally.

As with causes, consequence relations can be stated explicitly. In (1) below, the consequence is explicit, but the cause-consequence relationship needs to be inferred. However, the consequential relationship can be signalled linguistically, for example by a connective such as “and so”, as in (2).

1. Kate quit her job. She immediately started looking for a new one.
2. Kate quit her job, and so she immediately started looking for a new one.

When a consequence is not explicitly stated, it may nevertheless be implicit, just like a cause, particularly when it is not important for the development of the narrative. The way an event or state is described, and in particular the verb used, suggests which protagonist is the likely focus of the consequences of the event or state. For example, if *John frightened Mary*, it is unlikely that one can guess exactly what will follow as a consequence (e.g., “and so she avoided him for the rest of the evening”); what is more likely to be guessed is that it is *Mary* who suffered the consequences of being frightened.

Implicit causality has usually been associated with the causal directionality contained in the meanings of interpersonal verbs (Garvey & Caramazza, 1974; see Hartshorne, 2014, and Hartshorne, O’Donnell, and Tenenbaum, 2015, for a recent version of this hypothesis). Verbs that give rise to inferences that would assign the cause to the subject of a simple active sentence of the form *NP1 verb NP2,* and thus to the first noun phrase*,* are usually called NP1-biased. When the cause is assigned to the object, the verbs are referred to as NP2-biased. Consequentiality is likewise naturally associated with interpersonal verbs, and so the terms NP1-biased and NP2-biased must be used with caution. It is worth reiterating that the term “bias” is used because when implicit causality or consequentiality is measured by asking people to add explicit causes or consequences to statements containing interpersonal verbs or to make judgements about causality or consequentiality, the results are not completely consistent, but show a preponderance of responses favoring either the NP1 or the NP2.

As previously mentioned, the effects of implicit causality are well established, for example in timed reading tasks or plausibility judgments (Caramazza et al., 1977; McKoon et al., 1993; for a broader review, see Rudolph & Försterling, 1997). In particular, when the second clause in a sentence is consistent with the verb’s implicit causality bias, as in (3), then comprehension is faster than when the second clause in inconsistent with the verb’s implicit causality bias, as in (4)

3 Kate praised Liam because he had done well in his exams.
4 Kate praised Liam because she felt obliged to do so.

This effect is known in the literature as the *congruency* effect (e.g., Carreiras, Garnham & Oakhill, 1996; Garnham & Oakhill, 1985; Garnham, Oakhill & Cruttenden, 1992). A similar effect is found with implicit consequentiality (Stewart, Pickering, & Sanford, 1998a). One interesting set of questions arises because the same verb can have different causality and consequentiality biases, so it can be asked when those biases come into play in language processing, and how, if at all, they interact with each other.

In generating our implicit causality norms (Ferstl et al., 2011) we were able to consider a number of issues about implicit causality: its relation to verb semantic classes, thematic roles, and emotional valence, the possible roles of context and of differences in agentivity, which might also interact with the genders of the protagonists in the sentence fragments, and possibly with the gender of the participants, and its importance in fields other than psychology of language, such as linguistic pragmatics and social psychology. These considerations carry over to the study of implicit consequentiality. Because we have used an (almost) identical set of verbs in the current study, and because we wished to investigate the relation between implicit causality and implicit consequentiality, we have followed similar methods of data collection, processing and analysis in this study as in the previous one. Our norms will therefore be particularly useful where parallel sets of causality and consequentiality norms are required, and where sentence completion is the favored way of collecting the norms.

In relation to gender, we were interested in this factor in the causality norms (Ferstl et al., 2011) for two reasons. First, as is well established in the attribution theory literature, there are gender differences in attribution, both for people making attributions and for people identified as causes of particular behaviors (see, e.g., Simon & Feather, 1973; Swim & Sanna, 1996). Second, we noted in scoring the causality data that in some cases (e.g., for the verb “kill”) there was a difference in the ratio of NP1 to NP2 selections in causal completions depending on whether a male protagonist killed a female victim, or vice versa. Although consequences are different from causes, there may be similar gender effects on consequential selections, which would be simple to look for, and might be of interest in themselves.

There are many questions about implicit causality and consequentiality that are still under investigation. One such question is whether implicit causality has an early focusing effect (e.g., McDonald & MacWhinney, 1995; Long & De Ley, 2000; Koornneef & van Berkum, 2006; Pyykkönen & Järvikivi, 2010; Cozijn et al., 2011), or a later effect on clausal integration (Garnham, Traxler, Oakhill, & Gernsbacher, 1996; Stewart, Pickering, & Sanford, 2000). Recent evidence from comprehension tasks using event-related potentials (van Berkum et al., 2007) and the visual world paradigm (Pyykkönen & Järvikivi, 2010; Cozijn et al., 2011) seems to favor an early effect, either due to focusing or immediate integration. Similar effects can be found for implicit consequentiality (Garnham, Child, & Hutton, 2020), again raising the question of whether two biases, which may pull in different directions, operate together in language processing, or whether they only come into play when it is clear that either a cause or a consequence is being talked about.

To address these and related questions properly, a large set of verb norms for implicit consequentiality, paralleling those for implicit causality, is required.

## The present study

Studies of the effects of implicit causality and implicit consequentiality in sentence comprehension and production require normative data on specific verbs. Ferstl et al. (2011) provided implicit causality norms for over 300 two-person interpersonal verbs in English, which have enabled later researchers to replace their own intuitions, or norms for small numbers of verbs and rather few observations per verb. Examples of the use of small norming data sets include the first online reading study of implicit causality (Caramazza, Grober, Garvey, & Yates, 1977), which used norms for a set of 28 verbs collected by Garvey, Caramazza, and Yates (1974). In our own early online studies (Garnham, Oakhill, & Cruttenden, 1992) we also relied on these small-scale norms from Garvey, Caramazza, and colleagues. Stewart et al.’s (1998a) initial online studies of implicit consequentiality relied on their own corpus of 49 verbs.

To carry out replicable research on implicit consequentiality, and in particular of how it relates to implicit causality, a corresponding set of consequentiality norms is required. This consideration, and the fact that much of this work continues to be carried out in English, suggests that the present study is crucial. As previously mentioned, the new set of norms will also allow questions about the relation between implicit causality and implicit consequentiality to be answered. Thus, a sentence completion experiment was carried out using more or less the same set of 300+ verbs used by Ferstl et al. (2011).

As in Ferstl et al. (2011), we used a sentence completion task. This technique was used in the original Garvey and Caramazza (1974) paper on implicit causality. Participants provide an explicit consequence for an event for which the consequence, in the sense of the person most likely to be affected, is implicit at the end of the fragment. The sentence to be completed looks like example (5), where the linguistic signal “and so” is included to suggest that a consequence should be written. As in the previous study, we had protagonists of different genders and no subject noun phrase for the second clause, as that would pre-empt a choice of referent on the participant’s part.

5 Heather protected Craig and so …

To evaluate context effects and response strategies, we included the gender of the protagonist, as well as the gender of the participants in our analyses. The questions of interest were 1) whether male protagonists would be chosen more often as suffering the consequences of events than female protagonists, 2) whether such a difference would be modulated by the valence of the event, and 3) whether men and women would use different strategies for attributing consequentiality.

In addition, several reliability analyses were conducted to ensure comparability of our results with previously published data. We also looked at whether the four main semantic categories of verb showed the biases predicted by Crinean and Garnham (2006) and whether the consequentiality biases of the semantic classes were related to the causal biases in the way predicted in that paper. To recap, Crinean and Garnham predicted the following biases on the basis of a thematic roles analysis: AgPat (NP1 cause, NP2 consequence), AgEvo (NP2 cause, NP2 consequence), StimExp (NP1 cause, NP2 consequence), ExpStim (NP2 cause, NP1 consequence).

## Methods

### Verbs

Our starting point was the corpus of 305 past-tense verbs used in the Ferstl et al. (2011) study. The way that those verbs were selected is described in detail in that paper. After close consideration, two changes were made to this list. First “counseled” appeared in the list with US English spelling and was changed to the British English spelling “counselled”, as we would be testing British English participants. Second, although the paper says (2011: 127) that “disgruntled” was excluded, it appears in the supplementary material, with all the appropriate scores. However, since neither British (e.g., Oxford) nor US (e.g., Webster’s) online dictionaries include “disgruntle” as a verb, it was replaced by “bump”, which had been considered for the original list, but not included. We obtained valence data for “bump” as in the original study: using ratings from 12 independent participants on a 7-point scale for valence (ranging from −3: extremely negative, to +3: extremely positive). “Bump” was classified as an activity verb, with thematic role structure Agent-Patient (AgPat). As a reminder, the other categories were Agent-Evocator, Stimulus-Experiencer, and Experiencer-Stimulus (AgEvo, StimExp, ExpStim).

For all the verbs except “bumped”, length, emotional valence, semantic class, and thematic roles were carried over, after checks, from the Ferstl et al. (2011) study, and these factors are included in the analyses below. Word length was number of characters, including the space and preposition for 17 compound verbs (e.g., *apologize to*). We replaced the frequency counts from CELEX in Ferstl et al. (2011) with counts from the more recent, more extensive, and more relevant (to online processing) SUBTLEX-UK database (Van Heuven et al., 2014). Because we hypothesize (Crinean & Garnham, 2006) that implicit causality and implicit consequentiality are associated with verbs, not verb forms such as the past tense used in our study, we computed lemma frequencies. Note that we used past tense in the experimental passages because it is the most common form in narrative. Where possible, we used the measure “DomPoSLemmaTotalFreq” (total frequency for the lemma of the dominant part of speech) for the past tense form of the verbs. For some items, Verb was not the dominant part of speech for the “-ed” form (it was usually an adjective when it was not a verb). In these cases, we used the DomPoSLemmaTotalFreq associated with another verbal form (e.g., infinitival, “-s” or “-ing”) for which Verb was the dominant part of speech. In a few cases, where the dominant part of speech was not Verb for any of the verbal forms, we had to use information from the “AllPoSFreq” fields for forms that did occur as a verb (the verbs in question were “dumbfounded”, “like”, “nettled”, “troubled”, and we checked the infinitival, “-ed”, “-es”, and “-ing” forms of these verbs). Finally, two of our verbs had no related verbal entry in the database. For “abash” there were four occurrences in the corpus as an adjective (and 13 for “unabashed”), and for “jollify” the only related entry was “jollification”, with six occurrences as a noun. These verbs were recorded as having a frequency of 0.

For our 17 compound verbs, we searched the bigram file (SUBTLEX-UK_bigrams.csv) with the Unix tool “grep” to obtain the number of occurrences of the relevant compound forms. Again, we obtained a lemma-like measure by summing the infinitival, “-ed”, “-s”, and “-ing” forms. For “dream about”, we included “dreamt about” and well as “dreamed about”, the form used in the study, and for “take away” we included “taken away”, because the “-ed” forms of the other verbs would have included both actives and passives (e.g., “picked up”, “was picked up”).

For each verb we converted the count in the SUBTLEX-UK corpus to a Zipf score using the formula LOG10[(count + 1)/(201.336 + 0.159)]+3, provided by Van Heuven et al. (2014: 1180)—the denominator constants derive from the size of the corpus, and an estimate of how many words with an estimated frequency of 1 in a corpus of the same size did not occur in SUBTLEX-UK. In what follows, analyses that include frequency use these Zipf scores.

Descriptive statistics for the four verb classes and for the whole set of verbs are given in Table 1.

**Table 1:** Descriptive statistics for the whole verb corpus, and for each of the four linguistic categories

		Activity verbs		Psychological verbs		All verbs
		AgPat	AgEvo	StimExp	ExpStim	
	*N*	97	55	109	44	305
Word length (No. of letters)	*M*	7.7	8.4	8.2	7.8	8.0
	*sd*	2.1	1.8	1.8	1.9	1.9
	*range*	3 –14	4 – 13	4–13	5–13	3 – 14
Zipf frequency Scores (SUBTLEX)	*M*	4.27	3.34	3.47	4.09	3.79
	*sd*	0.87	1.04	1.09	0.98	1.07
	*range*	2.21 – 5.91	.70 – 5.87	.70 – 5.67	2.55 – 6.36	.70 – 6.36
Valence ratings	*M*	−.21	−.55	−.46	−.13	−.35
	*sd*	1.4	1.7	1.6	1.9	1.6
	*range*	−2.8 to +2.5	−2.7 to +2.7	−2.9 to + 2.5	−2.8 to + 2.5	−2.9 to +2.7
Bias score	*M*	−60.9	−74.7	−73.4	48.8	−52.1
	*sd*	34.7	14.0	23.9	42.8	51.3
	*range*	−93 to +71	−96 to −24	−95 to +87	−74 to +97	−96 to +97

As expected, word length and frequency were negatively correlated: *r* = −.39, *n* = 305, *p* < .001. As is well known, longer words tend to be less frequent. Emotional valence was determined as described above for “bump”. The valence ratings (*M* = −.35, *sd =* 1.6) were not correlated with length, but they were correlated with frequency (*r* = .21, *n =* 305, *p* < .001). There was a tendency for more common words to have more positive valence ratings.

One-way ANOVAs compared the four linguistic classes for frequency, length, and valence. The categories were well matched for valence, *F*(3, 301) = 0.99, but differed in frequency, *F*(3, 301) = 16.25, *p* < .001. For frequency, post hoc tests (Hochberg, and Gabriel, because of unequal Ns) showed that the following differences were significant: AgPat > AgEvo, *p* < .001; AgPat > StimExp, *p* < .001; AgEvo < ExpStim, *p* = .001, ExpStim > StimExp, *p* = .002 Gabriel, *p* = .003, Hochberg. There was also a tendency for a length to differ, *F*(3, 301) = 2.50, *p* = .06. AgPat and ExpStim verbs were slightly shorter than AgEvo and StimExp verbs. Because of these differences, length and frequency will be included in subsequent analyses as covariates.

### Experimental materials

To create a set of sentence fragments, Ferstl et al. (2011) needed common British English male and female forenames. They chose names from the “British names” section of the website “Baby Names World” (2008).^2^ Two native speakers of British English confirmed that 90 female and 90 male names were clearly unambiguous in gender and did not sound old-fashioned or bizarre. Beyond that number, they encountered names that were unusual, and might not have been unambiguously associated by their participants or ours with one gender or the other. Each name was, therefore, used in three or four sentence fragments.

One male and one female proper name were randomly assigned to each verb. For each verb, we created two sentence fragments, one with the male name in the NP1 position (“*M verbed F and so* …”), and one with the female name in the NP1 position (“*F verbed M and so*…”). For counterbalancing, one list was created with half of the sentences having a male NP1 and half a female NP1, and a second list was created by switching the proper names in each sentence fragment.

### Participants

One hundred and thirty-seven participants (107 Women, 28 Men, 2 other) took part in the study. Thirteen (3 male, 10 female) were excluded because their responses included at least 20 seriously deficient answers, so the data for 124 participants (97 female, 25 male) were included in the analyses reported. Excluded participants used tactics such as copying the same answer or a very similar answer (usually a very short one, e.g., “they were even”) on multiple trials, or entering a truncated answer, such as a pronoun by itself, or a dummy answer, such as “.” or “?”, so that the survey software would let them proceed to the next set of items. The age range of the participants included in the final analysis was from 17 to 34 years (1 under 18, 119 from 18–24, and 4 from 25–34). They were all first- or second-year undergraduate students at the University of Sussex who were native speakers of British English, and they received course credits for their participation.

### Procedure

We used a web-based version of the sentence completion task to assess the implicit consequentiality bias of the verbs, using Qualtrics online survey software (Qualtrics, Provo, UT, USA). Participants were contacted via the Sussex University SONA system (SONA Systems Ltd., Tallinn, Estonia) for participant recruitment, and if they satisfied the inclusion criterion (being a native speaker of British English), were sent a link to the Qualtrics questionnaire. Participants were assigned, by Qualtrics, to one of the two versions of the experiment alternately. Each participant completed a consent form, read the instructions, and provided simple demographic data (sex and age band) before proceeding to the main part of the study. The order of the sentence fragments was randomized individually for each participant by the Qualtrics software. The participants were instructed to type a sensible completion for each sentence fragment, similar to the examples provided to them (e.g., “John injured Mary and so she had to go to the hospital”). They were also instructed to answer spontaneously and complete each sentence at once without going back and revising previous answers. There was no time pressure on participants, and they could proceed at their own speed. However, the sentence fragments were divided into six blocks for each participant, and it was suggested that ends of blocks were sensible places to take a break. Qualtrics did not allow a participant to proceed if any response was completely blank; so, in this sense, there were no completely missing responses (but see below, under Coding). After the completion of the questionnaire, the participants were notified that their task was over and they had to press the “Submit” button in order to send their data to the server. The completion of the entire questionnaire lasted for 40 minutes or more, depending on the participant’s response speed and the number and length of breaks taken. The time recorded by Qualtrics was from first accessing the questionnaire and final submission of the data, which could be considerably longer.

### Coding

For each response, we coded whether it referred to the first noun phrase in the sentence fragment (NP1) or the second (NP2). Other, excluded types of response included reference to both characters (using a plural pronoun such as “they”, a conjoined pair of names such as “John and Mary”, or a word or phrase such as “both”—3770 or 10% of responses), reference to another person, an indefinite reference (e.g., “someone”), use of “it”, which might be a reference to an event or non-referential (e.g., “Russell avoided Joanna and so it was awkward”) (917 or 2.4% of responses), ambiguous references, uninterpretable continuations, and fillers such as “.” and “?”, that had to be entered to allow the participant to complete the questionnaire (81 or 0.21% of responses). With consequential continuations using “and so”, it is also possible to produce just a verb phrase (VP) which is interpreted as conjoined with the VP of the presented fragment. Such VPs should be interpreted as having the same subject as the fragment, and hence have an NP1 reference (e.g., “Sean disdained Karen and so….did not listen to what she had to say”—2735 examples, 7.2%). Nevertheless, the content of a minority of VP continuations could only be interpreted as containing a reference to the NP2 (e.g., “Edgar startled Angela and so….shrieked in horror”—160 examples, 0.2%). In the first author (a native British English speaker)’s dialect such continuations are ungrammatical. Nevertheless, we also reported continuations of this kind in another study (Garnham & Ivic, 2017), and they were scored as NP2 references, so we included them here as contributing to NP2 bias. We also reclassified some continuations on the basis of the underlying meaning; for example, in “Chloe intimidated Ewan and so when she approached him, his face went red”, the first reference after “and so” is to Zoe (“she”), but the consequence of the intimidation was that Ewan’s face when red, so an NP2 consequence.

Initial scoring was carried out using a semi-automatic procedure in Microsoft Excel. All responses that started with “he” or “she” or with one of the two names in the fragment were initially scored as NP1 or NP2 completions using information about the position of the male and female names in the fragment (28,837 responses, 76.2%). The responses were then checked manually, to reclassify to NP1 or NP2 where necessary, based on underlying meaning (see above), and to check that those beginning with a name did not have a conjoined subject NP (e.g., “Heather and Craig…” as a continuation for example 5). The remaining completions that were not processed automatically (8983 responses, 23.8%) were scored by the second and third authors with instructions from the first author. The second and third authors checked a proportion of each other’s responses, and all remaining problematic cases were resolved in a discussion amongst all three authors.

In the final classification, 87% of the continuations were either NP1 or NP2, and the other 13% were excluded. For each verb, its bias score was defined as the difference between the number of NP1 and NP2 responses, as a proportion of the total number of valid responses [i.e., bias = 100 × (noNP1 – noNP2)/(noNP1 + noNP2), with noNP1 being the number of NP1 continuations, and noNP2 being the number of NP2 continuations]. Bias scores, therefore, varied between 100 (all relevant continuations attributed the consequence to NP1), and −100 (all relevant continuations were NP2 consequences). A bias score of 0 reflects an equal number of NP1 and NP2 continuations. Excluded responses did not figure in the calculation.

The consequentiality scores, together with number of NP1 and NP2 completions, plus verb class information and causality bias scores from Ferstl et al. (2011) are provided in an Appendix. A more complete set of scores for the 305 verbs is available in the University of Sussex Research Data Repository as supplementary material (10.25377/sussex.c.5082122). In this more complete dataset, the numbers of NP1 and NP2 completions are presented separately for male and female participants, and according to whether the first noun phrase was male or female. In addition, lexical and semantic features, including frequency (SUBTLEX counts and Zipf scores), length, valence ratings, and verb class, are also provided.

## Results

Across participants, 12.6% (4768) of the responses were not classifiable as NP1 or NP2 (*m* = 38.45, *sd =* 20.60, range: 4–127). Focusing on the responses of interest, 20.4% of the total were NP1 continuations (*m* = 62.29, *sd* = 25.37, range: 78–227), and 67.0% NP2 continuations (m = 204.26, *sd* = 31.12, range: 48–284), indicating that all participants used a variety of responses. NP2 continuations were more frequent than NP1 continuations, as three of the four verb classes (261/305 verbs) were predicted to have NP2 consequentiality biases (see section “Gender” for the full statistical analysis by participants).

Across verbs, the bias scores were widely distributed, but with a strong overall tendency to NP2 bias, which was predicted for three out of the four classes of verbs (*M =* −52.1, *sd =* 51.3, range: −96 to +97). This preference for NP2 continuations was highly significant in the analysis by items, *t*(301) = 200.18, *p* < .001.. Post hoc analyses (Hochberg and Gabriel, see above) suggested that the only classes that did not differ in overall bias were AgPat and StimExp.

Assuming a random binomial distribution of NP1 and NP2 continuations with 124 observations, and probabilities of 0.5 for NP1 and NP2 continuations, the mean would be 62 continuations of each kind and the standard deviation 5.57. With bias scores ranging from −100 to +100, scores below −18 and above 18 are significant at the 5% level and ±21 at the 1% level. According to the 1% criterion, a large number of verbs in the corpus show a significant bias towards either NP1 (*n* = 41) or NP2 (*n* = 250). Thirty of the NP1 verbs and 228 of the NP2 verbs even met the very strict criterion of a bias score above 50 or below −50.

### Reliability

To confirm that the continuations collected using our web-based questionnaire replicated previous results, we compared our bias scores to previously published normative data.

Au (1986, Experiment 1) collected consequential (“so”) completions for 48 verbs, 12 each from our four semantic classes (she called Agent-Patient and Agent-Evocator Action-Agent and Action-Patient, respectively). For each verb, she calculated the percentage of responses referring to one role (Experiencer or Patient). She also collected data for active and passive main clauses. We used her data for actives, as they were more directly comparable with our own. For comparison with our own scores, which were positive for NP1-biased verbs, we subtracted the % Experiencer scores from 100 for Stimulus-Experience verbs and the % Patient scores from 100 for both classes of Action Verb to get the percentage of references to the NP1 (it is implied, but not directly stated, that the percentages were calculated on completions with clear NP1 or NP2 references only). For the set of 48 verbs, the Pearson product-moment correlation with the bias scores collected in the present study was *r* = .95, *n* = 48*, p* < .001. There was one qualitative difference between the two sets of results. *Esteem*, which was relatively weakly NP1-biased (65%) in the Au norms, was even more weakly NP2-biased in the current set. In addition, *dread* was considerably more strongly biased in the present data, and Au had a number of verbs with a 100% bias, reflecting the fact that she had 20 or fewer completions per verb.

Stewart et al. (1998b, see Crinean & Garnham, 2006) conducted a sentence completion study using 49 verbs and 32 participants For these 49 verbs the correlation between their consequentiality scores (using the same formula as defined above, computed from the data presented in Crinean & Garnham, 2006: 647) and scores from the present web-based questionnaire was again very high (*r =* .96, *n* = 49, *p* < .001). Note, that these verbs had been selected to have strong causality biases, though that does not necessarily imply they would have strong consequentiality biases (Stewart et al., 1998b). There were a very small number of notable differences. *Deplored*, which was one of the less strongly NP1-biased ES verbs for Stewart et al., was very slightly NP2-biased in our dataset, and *noticed*, which was very weakly NP1-biased for Stewart et al., was more strongly biased in our dataset.

Hartshorne, O’Donnell, and Tenenbaum (2015) collected “Result” norms using items of a different kind containing nonce words in explicitly provided results (= consequences, for example, “Because Sally VERBed Mary, she daxed”), and a different task (“Who do you think daxed?”). Their items included 165 of the same verbs that we used (10 other verbs were in common but changed their meaning and likely their consequential bias by the addition of a particle, e.g., “feared” in our norms vs. “feared for” in theirs). From their data, we calculated the number of NP1 responses out of the total number of reported responses. It is not clear whether all reported responses had a reference that was clearly to NP1 or clearly to NP2, though that would be a sensible way of presenting the data. The correlation between their results and ours was *r* = .85*, n* = 165*, p* < .001. Differences in materials and methodology may explain the slightly lower correlation than with the Au and Stewart et al. norms.

### Length and frequency

The large number of items allows us to evaluate the influence of lexical features. The bias scores were correlated with the word frequency. High-frequency verbs elicited more NP1 continuations than verbs lower in frequency (*r* = .22, *n* = 305, *p* < .001, though overall bias scores were predominantly negative, indicating mainly NP2 continuations, and the correlation was negative for all four verb classes, ruling out an explanation in terms of consequentiality). This pattern is the opposite of that found in the causality bias norms, because the majority of verbs switched bias in the consequentiality data presented in this paper. There was also a significant correlation between word length and bias (*r* = −.17, *p* < .01), which again switched sign for related reasons. Longer words had more negative (NP2) bias scores, and since bias scores were predominately negative, the pattern was that longer words tended to have more extreme NP2 biases.

### Thematic roles and semantic class

Crinean and Garnham (2006) argued that, on the basis of semantic analysis, StimExp and ExpStim verbs have the Stimulus as the implicit cause and the Experiencer as the implicit consequence. AgPat verbs have Agent as implicit cause and Patient as implicit consequence, and AgEvo verbs have Evocator in both roles. These patterns held in the norms of Stewart et al. (1998b), but those norms included only verbs known to have strong causal biases. Empirically, it is well established that action verbs show a more varied pattern of implicit causality biases that mental state verbs. Although the Agent brings about the action, there are many other factors, including the Patient or, especially, the Evocator (for AgEvo verbs), that may influence the Agent. AgEvo verbs give relatively consistent results, as the Evocator has some of the properties of a Stimulus (Crinean & Garnham, 2006), but AgPat do not (e.g., Rudolph & Försterling, 1997). Stimuli more straightforwardly bring about experiences, and if those stimuli are people, there are many things about those Stimuli that may bring about the experiences, without considering other causes.

**Fig. 1:**
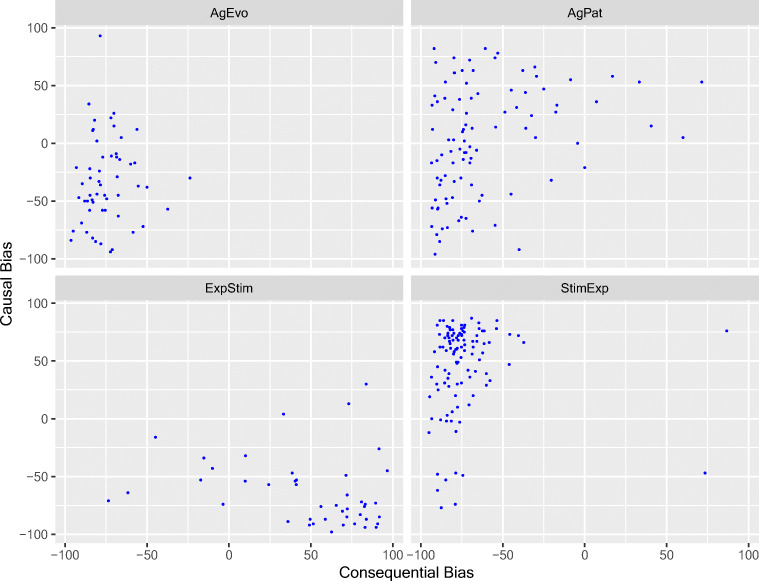
Scatterplots of implicit causality bias vs. implicit consequentiality bias for the four classes of verb: AgPat, AgEvo, StimExp, and ExpStim

There were 304 verbs in common between the causality and consequentiality norms. *Disgruntle* appeared only in the causality norms. It was classified as StimExp and had a positive (NP1, 58%) causality bias, as expected for a StimExp verb. *Bump* occurred only in the consequentiality norms. It was classified as AgPat and had a negative (NP2, −31%) consequentiality bias, again as expected. Table 2 shows the pattern of results across the two sets of norms, and Fig. 1 shows scatterplots of causality bias vs. consequentiality bias for the four classes of verbs. As suggested above, the action verbs, and AgPat in particular, conform less strongly to the pattern identified by Crinean and Garnham (2006) than the other three classes.

**Table 2:** Classification of 304 verbs (+ “disgruntle” and “bump”) by semantic class, causality, and consequentiality (bias > 0 = NP1; bias < 0 = NP2)

	AgPat	AgEvo	StimExp	ExpStim
Number of verbs	96 + bump	55	109 + disgruntle	44
NP1 causality	51 + bump	11	94 + disgruntle	3
NP2 causality	44*	44	15	41
NP1 consequentiality	7	0	2	37
NP2 consequentiality	89	55	107	7
Predicted pattern	NP1 cause NP2 conseq.	NP2 cause NP2 conseq.	NP1 cause NP2 conseq.	NP2 cause NP1 conseq.
Number	45/96	44/55	92/109	34/44

*One AgPat verb had a measured bias of exactly 0

**Fig. 2:**
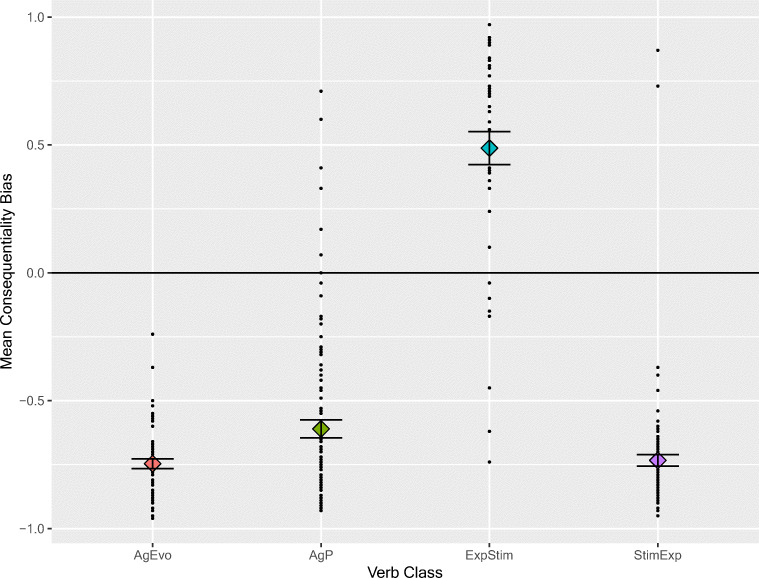
Individual bias scores for items in the four linguistic categories (black dots) and means (filled diamonds). The short horizontal lines show one standard error above and below the mean

Figure 2 shows the mean bias score for each of the four verb types. As expected, the bias scores differed considerably for the categories: AgEvo, AgPat, and StimExp verbs elicited more NP2 continuations, and ExpStim verbs more NP1 continuations. An ANCOVA was conducted with Semantic Category as a factor with four levels, controlling for length, frequency, and valence. In contrast to the causality norms, it did not make sense to characterize Semantic Category as a 2 × 2, with activity verb vs. psychological verb (i.e., AgPat/AgEvo vs. ExpStim/StimExp), and expected NP1 causality vs. expected NP2 causality (i.e., AgPat/StimExp vs. AgEvo/ExpStim) as factors. For the covariates, the effects were word length, [*F*(1, 298) = 5.58, *p* = .019], frequency [*F*(1, 298) = 3.70, *p* = .055], and valence [*F*(1, 298) = 2.35, *p* = .127].

Controlling for these factors, there was a highly significant effect of Semantic Category, *F*(3, 298) = 197.3, *p* < .001. The means for the four categories (*sd* in parentheses) were AgPat −61 (35), AgEvo −75 (14), ExpStim 49 (43), and StimExp −73 (24). Bonferroni-corrected *t* tests showed that all the differences except that between AgEvo and StimExp were significant (see Table 3).

**Table 3:** Bonferroni-corrected pairwise comparisons of consequentiality differences among the four classes of verb

Comp	Levine	*t*	df	Sig.	Diff.	Bonf.
AgPat vs. AgEvo	23.179	3.448	138.680	.001	.13782	/6
AgPat vs. StimExp	21.467	2.970	167.631	.003	.124798	/6
AgPat vs. ExpStim	2.737 ns	16.125	139	<.001	1.09652	/6
AgEvo vs. StimExp	.244	.373	162	Ns	.013024	/6
AgEvo vs. ExpStim	35.146	18.342	50.359	<.001	1.23434	/6
StimExp vs. ExpStim	27.996	17.820	54.145	<.001	1.22131	/6

### Gender

**Fig. 3:**
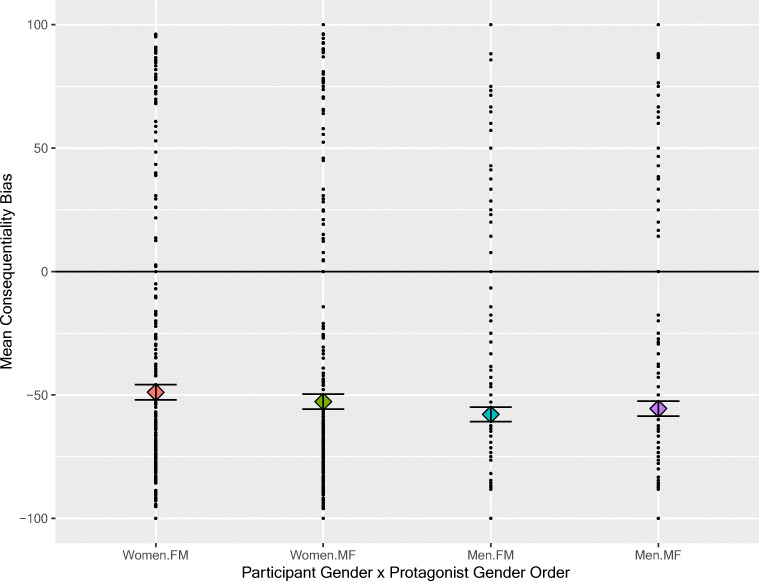
Differential effects of the gender of the names in the sentence fragment (FM = female-male, MF = male-female) on the continuations chosen by women and men. More negative scores indicate stronger NP2 biases. Individual bias scores are shown as black dots and means as filled diamonds. The short horizontal lines show one standard error above and below the mean

To evaluate the effects of the gender of the participants and of the protagonists in the sentence fragments, an analysis by participants on consequentiality bias scores was conducted. The ANOVA included the within-participant factor Referent Gender Order (FM vs. MF) and the between-participant factor Participant Gender (women vs. men—because there was only one participant declaring their gender as “other” in each version of the experiment, it was not possible to include “Other” as a level of this factor). Positive (NP) consequentiality biases favor female referents for the FM order and male referents for the MF order. A main effect of Gender Order would have indicated an overall preference for continuations attributing the consequence to either the female character in the sentence fragment or the male character, but the effect was not significant (*p* > .05). The interaction between Participant Gender and Order of Referents was highly significant, *F*(1, 120) = 12.47, *p* < .001). Female participants tended to favor reference to female characters and male participants to male characters (see Fig. 3, an effect of +2%).

For an item analysis of these gender effects, we conducted a 2 × 2 within-item ANCOVA, controlling for the factors valence, frequency, and length. This analysis confirmed the analysis by participants. There were significant interactions of Participant Gender and whether the sentence had a female protagonist followed by a male or a male followed by a female, *F*(1, 301) = 4.63, *p* < .05, and a three-way interaction of those factors and length, *F*(1, 301) = 9.73, *p* < .01. As noted above, the two-way interaction indicates a preference of participants to refer to protagonists of their own gender—an effect of about 4% for women and 2% for men in both the raw means and in the expected marginal means from the ANCOVA. Of the covariates, only frequency was significant, *F*(1, 301) = 8.35, *p* = .01.

Table 4 displays the individual verbs that were particularly sensitive to gender differences, i.e., those verbs for which the bias scores differed greatly (by more than 0.3 on a scale from −1 to +1), depending on whether NP1 was male or female. As can be seen, the verbs eliciting more male continuations tend to be negative in valence, whereas verbs that are more likely to elicit a female continuation have more positive valence ratings.

**Table 4:** Verbs that showed exceptionally large gender effects. The table shows verb class, valence ratings, bias scores (with negative values indicating NP2 bias, positive values NP1 bias; see text for formula), and gender effect. The gender effect is the difference in bias scores when NP1 was male and when NP1 was female. Positive scores indicate a greater tendency to refer to male characters, negative scores a greater tendency to refer to female characters

Verb	Verb class	Valence rating	Overall bias score	Gender effect
Male-biased				
Calmed	StimExp	1.6	−85	30
Debated	AgPat	−.6	−18	38
Disdained	ExpStim	−2.1	−10	39
Escorted	AgPat	0	−69	34
Fascinated	StimExp	1.6	−54	37
Killed	AgPat	−2.8	+60	44
Met	AgPat	.8	+71	50
Female-biased				
Carried	AgPat	.1	−40	−52
Enthralled	StimExp	.9	−40	−65
Harmed	AgPat	−2.0	−72	−34
Incensed	StimExp	−1.1	−62	−32
Left	AgPat	−1.5	−73	−36
Married	AgPat	2.4	+33	−56
Noticed	ExpStim	.6	+49	−33
Pardoned	AgEvo	.1	−50	−81
Shadowed	AgPat	−.7	+17	−33
Took away	AgPat	−1.5	−64	−43
Tracked	AgPat	−.3	+07	−38
Trailed	AgPat	−.3	−30	−31
Welcomed	AgEvo	1.2	−57	−49

### Emotional valence

Unlike in the causality norms (Ferstl et al., 2011), there was no effect of valence nor any interaction with the other factors in the ANCOVA. Relatedly, there was no simple correlation between valence and consequentiality bias scores (*r* = .012, *n* = 305, n.s.).

## Discussion

The study provides normative data on implicit verb consequentiality in English for the same set of interpersonal verbs for which Ferstl et al. (2011) provided implicit causality norms. To elicit consequences, we used the same sentence completion technique, but asked participants to complete sentence fragments ending with the connective “and so”, rather than “because”. The results replicate the small number of previous studies on consequentiality, and allow for a detailed examination of the hypotheses of Crinean and Garnham (2006) about the relation between implicit causality and implicit consequentiality for the four classes of verbs standardly recognized in the implicit causality literature: Agent-Patient (AgPat), Agent-Evocator (AgEvo), Stimulus-Experiencer (StimExp), and Experiencer-Stimulus (ExpStim). With over 300 verbs, we showed that a majority of these verbs exhibit a clear bias in a standard sentence completion test, to either NP1 or NP2 consequentiality. Indeed, consequentiality biases were more consistent by Verb Class than causality biases, which, particularly for AgPat verbs, were somewhat variable. The majority of verbs in the four classes showed the consequentiality biases expected on the basis of a thematic roles analysis (AgPat—NP2, Patient; AgEvo—NP2, Evocator; StimExp—NP2, Experiencer; ExpStim—NP1, Experiencer). For consequentiality, as for causality, our norms show a wide range of biases spread over the whole range (see Fig. 1), though for consequentiality, unlike causality, there is an overall tendency to NP2 bias. These results are based on a large group of respondents, each asked to provide completions for every verb, and should, therefore, provide accurate estimates of the biases of individual verbs. They also provide information that closely parallels our causality information for the same verbs and will be particularly useful in studies in which causality and consequentiality information for the same verbs is needed.

When the same verbs were used, our data largely replicate the results of previous normative studies (Au, 1986; Stewart et al., 1998b; Hartshorne, O’Donnell, & Tenenbaum, 2015).

As we noted in the causality norms paper, it is encouraging that online data collection with partly automated scoring procedures produces similar results to previous “pencil and paper” studies. However, we have noted several places in which care must be taken in using automated procedures. While we have tried to ensure that we have coded these cases correctly, they are, in fact, relatively rare. So, with a large dataset, they can have only small effect on measured norms.

We have followed much of the psycholinguistic literature in using the four-way classification of verbs into the classes AgPat, AgEvo, ExpStim, and StimExp. Harsthorne (e.g., Hartshorne et al., 2015) has argued for a somewhat finer-grained analysis, based on the verb categories identified by Levin (1993) and used in the VerbNet project (Kipper, Korhonen, Ryant, & Palmer, 2008). However, it is unclear from the data presented by Hartshorne et al. (2015, Figures 3 and 5) that this analysis provides additional insights, particularly in the case of implicit consequentiality, where most verbs show an NP2 consequentiality bias. In the framework adopted here, within the psychological verbs, ExpStim and StimExp verbs show different biases, as the consequences usually fall on the Experiencer, who is NP1 for ExpStim verbs and NP2 for StimExp verbs. For the activity verbs, both subclasses showed an NP2 bias, as consequences usually fall on the Patient for AgPat verbs and on the Evocator for AgEvo verbs.

The fact that AgEvo verbs, unlike the other three categories, do not show a switch in bias between causality and consequentiality relates to the observation by Crinean and Garnham (2006) that AgEvo verbs often have a psychological component to their meaning. Thus, they effectively have an ExpStim component, though the “Experiencer” also performs an (evoked) action, and so has the properties of an agent. However, the NP2 in its Stimulus role is often identified as the implicit cause, rather than the Agent. For consequences, the fact that the Evocator is acted upon, gives it a Patient role that is associated with consequences.

As in the causality study, we examined effects of lexical features that are known to influence processing in other domains (e.g., lexical access or reading times). Furthermore, we found influences of these factors in the sentence completion study of causality (Ferstl et al., 2011). In this study of consequentiality, length influenced the direction of implicit consequentiality. Given that most verbs switched bias from the causality study, the effect of length also switched. Longer words tend to show stronger NP2 bias. Similarly, SUBTLEX frequency had the opposite effect from in the causality norms. So, given the relation between length and frequency, we found that less frequent, longer words elicited more NP2 continuations. This result is not readily interpretable and might depend on the particular selection of verbs. However, lexical factors are undoubtedly important in online studies on verb causality. Shorter words and more frequent words are read faster, they are accessed more quickly, and they are subjectively more familiar. Thus, it is crucial to control for these factors. Given that the present corpus contains many verbs with very strong biases towards either NP1 or NP2 (250+ with biases > 50 or < −50), it becomes possible to select subsets to match or manipulate these lexical features.

In addition to the lexical features mentioned above, we also present ratings of the verbs’ emotional valence. This factor has been studied particularly in social psychological studies of causality (Corrigan, 2001; Semin & Marsman, 1994). However, although we found effects of valence in our causality study, we did not find such effects here. These different effects may repay more systematic study, including investigations of how or whether they are manifested in online comprehension. Our normative data will be helpful in selecting appropriate verbs for such studies.

As in the causality study, we were also interested in effects of gender, both effects of the genders of the participants in the interpersonal events, and those of the gender of the participants. Furthermore, there may be interactions between these two types of gender effects. Our findings for consequentiality were somewhat more straightforward than those for causality. There were verbs that showed strong preferences for reference to females over males or vice versa (see Table 3). For these verbs, it made a difference whether the male or the female protagonist was mentioned first, independent of the specific direction of the bias. However, for consequentiality, unlike what we reported for causality, there were no obvious systematic differences between the two sets. This difference between causes and consequences may reflect the differing importance of ascribing causes and identifying consequences in society.

For participants, we found a small but significant tendency for women to prefer references to the first NP (NP1) and another small but significant tendency for people to prefer to refer to protagonists of their own gender. Unfortunately, our ability to investigate participant gender effects in this study was hampered by the predominance of female participants—there was a much greater gender imbalance in this study than in the causality study.

Our corpus of normative data on implicit consequentiality biases neatly complements our previous implicit causality corpus, and should, either by itself or in conjunction with the causality corpus, be useful in a range of studies in psycholinguistics and social psychology and, no doubt, other areas of psychology. The two corpora provide parallel data on over 300 verbs, and for each verb, reliable data based on the responses of around 100 respondents. As we noted in connection with the causality norms, studies that require a large number of different items, such as ERP and fMRI work, will benefit particularly, as will experiments requiring correlational analysis. Good estimates of individual verb biases for a large number of items will eliminate some noise from the data collected in such studies.

In addition, the corpus can be useful in a variety of applications beyond psycholinguistics. In particular, studies of pragmatic knowledge, social interactions, and interpersonal relations can benefit from a corpus that allows control of lexical properties of stimuli. Besides the intentional manipulation of implicit verb causality and consequentiality in such studies, the corpus can also help to avoid unwanted or confounding biases by selecting neutral verbs. For example, we recently conducted a study on the processing of gender stereotype information, as it is present in culturally defined nouns (e.g., “kindergarten teacher” is more likely to be interpreted as a woman). The availability of a large number of neutral verbs facilitated this study.

Implicit consequentiality and implicit causality remain interesting research areas with many open questions. The present corpus could facilitate studies of lexical and semantic representation in psycholinguistics, as well as studies of interpersonal relations and cultural norms in social psychology, particularly where consequentiality and causality are studied together.

## Appendix

The 305 verbs with the sematic categories, total NP1 and NP2 responses (out of 124), and consequential bias scores. The causal bias scores from Ferstl et al. (2011) are included for comparison.

**Table:**

Verb	Semantic category	Total NP1 responses	Total NP2 responses	Bias Scores	
				Conseq. bias score	Causal bias score (Ferstl et al.)
abandoned	AgPat	4	113	−93	33
abashed	StimExp	6	108	−89	25
abhorred	ExpStim	64	39	24	−57
acclaimed	AgEvo	13	93	−75	−58
accompanied	AgPat	6	72	−85	−48
accused	AgEvo	10	93	−81	2
admired	ExpStim	101	18	70	−92
admonished	AgPat	7	107	−88	−32
adored	ExpStim	98	9	83	−74
advised	AgPat	9	112	−85	−28
affected	StimExp	22	88	−60	29
affronted	StimExp	15	87	−71	12
aggravated	StimExp	9	107	−84	59
agitated	StimExp	7	113	−88	85
alarmed	StimExp	12	106	−80	58
alienated	AgPat	5	112	−91	41
amazed	StimExp	15	99	−74	68
amused	StimExp	9	94	−83	67
angered	StimExp	11	102	−81	85
annoyed	StimExp	10	104	−82	79
answered	AgPat	11	78	−75	−64
antagonized	StimExp	9	103	−84	80
apologized to	AgEvo	9	75	−79	93
appalled	StimExp	27	90	−54	78
appeased	StimExp	15	79	−68	20
applauded	AgEvo	2	108	−96	−84
appreciated	ExpStim	83	28	50	−87
approached	AgPat	14	77	−69	39
astonished	StimExp	22	101	−64	51
astounded	StimExp	19	100	−68	62
attracted	StimExp	13	71	−69	87
avoided	AgPat	21	71	−54	14
baffled	StimExp	12	108	−80	56
banished	AgPat	4	112	−93	−56
battled	AgPat	24	40	−25	47
beguiled	StimExp	21	84	−60	39
believed	ExpStim	73	31	40	−54
betrayed	AgPat	10	89	−80	74
bewildered	StimExp	13	107	−78	49
blamed	AgEvo	40	65	−24	−30
blessed	AgEvo	4	112	−93	−21
bored	StimExp	15	106	−75	73
bothered	StimExp	16	104	−73	59
bugged	StimExp	15	105	−75	72
bumped	AgPat	41	78	−31	
called	AgPat	3	71	−92	82
calmed	StimExp	8	97	−85	−53
calmed down	AgPat	5	98	−90	−79
captivated	StimExp	14	99	−75	78
caressed	AgPat	8	101	−85	39
carried	AgPat	33	77	−40	−92
castigated	AgEvo	14	102	−76	−45
caught	AgPat	30	79	−45	−44
cautioned	AgPat	7	114	−88	−36
celebrated	AgEvo	20	64	−52	−72
censured	AgEvo	13	101	−77	−58
charmed	StimExp	5	95	−90	81
chased	AgPat	11	97	−80	−33
chastened	AgEvo	9	108	−85	−30
chastized	AgEvo	10	107	−83	−51
cheated	AgPat	14	97	−75	63
cheered	StimExp	6	112	−90	−48
cherished	ExpStim	72	30	41	−53
chided	AgEvo	6	108	−89	−35
chilled	StimExp	8	102	−85	31
comforted	StimExp	7	105	−88	−77
commended	AgEvo	10	109	−83	−82
compensated	AgPat	14	88	−73	16
complemented	AgPat	6	108	−89	−56
complimented	AgEvo	5	114	−92	−47
concerned	StimExp	15	106	−75	81
condemned	AgEvo	19	98	−68	−63
confessed to	AgPat	24	82	−55	74
confided in	AgPat	35	65	−30	5
confounded	StimExp	16	91	−70	36
confused	StimExp	13	109	−79	60
congratulated	AgEvo	14	87	−72	−94
consoled	StimExp	12	102	−79	−74
consulted	AgPat	33	70	−36	13
corrected	AgPat	8	114	−87	−74
corrupted	AgPat	13	97	−76	38
counseled	AgPat	14	107	−77	−67
courted	AgPat	22	31	−17	33
criticized	AgEvo	9	109	−85	−45
cuddled	AgPat	6	88	−87	−10
dated	AgPat	26	11	41	15
daunted	StimExp	10	112	−84	72
debated with	AgPat	14	20	−18	27
deceived	AgPat	19	100	−68	63
decried	AgEvo	14	85	−72	−11
defamed	AgEvo	8	102	−85	34
defied	AgPat	30	87	−49	27
delighted	StimExp	7	94	−86	85
denigrated	AgEvo	10	106	−83	12
denounced	AgEvo	11	91	−78	−36
deplored	ExpStim	47	64	−15	−34
deprecated	AgEvo	17	91	−69	−12
derided	AgEvo	11	94	−79	−24
deserted	AgPat	6	113	−90	36
despised	ExpStim	91	8	84	−87
detested	ExpStim	93	15	72	−78
disappointed	StimExp	32	86	−46	73
discouraged	StimExp	4	119	−93	36
disdained	ExpStim	49	60	−10	−43
disliked	ExpStim	81	21	59	−87
disobeyed	AgPat	53	63	−9	55
disparaged	AgEvo	25	89	−56	12
distracted	StimExp	14	100	−75	53
distressed	StimExp	13	107	−78	60
distrusted	ExpStim	91	19	65	−75
divorced	AgPat	34	34	0	−21
dominated	AgPat	12	108	−80	3
dreaded	ExpStim	108	6	89	−73
dreamed about	ExpStim	113	10	84	30
dumbfounded	StimExp	17	101	−71	42
echoed	AgPat	14	80	−70	72
embraced	AgPat	8	74	−80	29
employed	AgPat	18	96	−68	−76
encouraged	StimExp	3	118	−95	−12
enlightened	StimExp	4	116	−93	0
enlivened	StimExp	9	96	−83	39
enraged	StimExp	12	98	−78	70
enthralled	StimExp	28	66	−40	72
enticed	StimExp	8	101	−85	70
entranced	StimExp	15	102	−74	76
envied	ExpStim	108	10	83	−94
escorted	AgPat	15	82	−69	−36
esteemed	ExpStim	48	68	−17	−53
exalted	AgPat	16	91	−70	−17
exasperated	StimExp	12	107	−80	74
excited	StimExp	9	85	−81	72
excused	AgEvo	7	110	−88	−50
exhausted	StimExp	10	102	−82	65
exhilarated	StimExp	6	83	−87	62
fancied	ExpStim	112	6	90	−94
fascinated	StimExp	26	86	−54	85
favoured	ExpStim	68	32	36	−89
fazed	StimExp	10	107	−83	28
feared	ExpStim	117	5	92	−85
fed	AgPat	7	115	−89	−85
filmed	AgPat	16	91	−70	−3
flabbergasted	StimExp	11	110	−82	61
flattered	StimExp	9	113	−85	42
floored	AgPat	18	97	−69	13
followed	AgPat	32	84	−45	46
fooled	AgPat	15	103	−75	10
forgave	AgEvo	6	29	−66	5
forgot	ExpStim	32	84	−45	−16
fought	AgPat	25	49	−32	24
freed	AgPat	9	104	−84	−52
frightened	StimExp	14	109	−77	68
frustrated	StimExp	15	99	−74	79
galled	StimExp	12	96	−78	30
gladdened	StimExp	12	93	−77	72
grabbed	AgPat	13	96	−76	−5
grazed	AgPat	37	79	−36	44
greeted	AgPat	12	75	−72	−8
grieved	StimExp	98	15	73	−47
guided	AgPat	9	102	−84	−73
hailed	AgEvo	18	93	−68	−45
harassed	StimExp	20	100	−67	41
harmed	AgPat	16	99	−72	52
hated	ExpStim	91	12	77	−91
haunted	StimExp	13	110	−79	20
helped	AgPat	5	107	−91	−49
hired	AgPat	16	100	−72	−65
hit	AgPat	15	101	−74	−14
honoured	AgEvo	36	79	−37	−57
hugged	AgPat	13	87	−74	12
hurt	StimExp	31	84	−46	47
idolized	ExpStim	105	17	72	−66
incensed	StimExp	20	86	−62	57
infuriated	StimExp	15	98	−73	75
inspired	StimExp	21	97	−64	78
instructed	AgPat	4	118	−93	−17
insulted	StimExp	11	104	−81	6
interrupted	AgPat	10	108	−83	3
intimidated	StimExp	7	116	−89	73
intrigued	StimExp	22	95	−62	76
invigorated	StimExp	11	86	−77	49
irritated	StimExp	15	97	−73	81
jollified	StimExp	8	78	−81	−2
jolted	StimExp	14	104	−76	−3
killed	AgPat	92	23	60	5
kissed	AgPat	10	88	−80	61
lauded	AgEvo	25	87	−55	−37
laughed at	AgPat	5	110	−91	−96
led	AgPat	11	79	−76	−30
left	AgPat	15	98	−73	2
lied to	AgPat	27	88	−53	78
liked	ExpStim	103	5	91	−91
loathed	ExpStim	92	15	72	−85
loved	ExpStim	77	14	69	−80
maddened	StimExp	11	102	−81	77
married	AgPat	16	8	33	53
mesmerised	StimExp	20	97	−66	72
met	AgPat	24	4	71	53
missed	ExpStim	116	2	97	−45
mocked	AgEvo	12	104	−79	−33
mollified	StimExp	9	106	−84	−2
mourned	ExpStim	104	11	81	−72
moved	StimExp	12	100	−79	−11
nettled	StimExp	14	99	−75	31
noticed	ExpStim	85	29	49	−92
nuzzled	AgPat	10	87	−79	61
ordered around	AgPat	9	111	−85	53
pacified	StimExp	15	102	−74	−49
pained	StimExp	13	104	−78	61
pardoned	AgEvo	24	72	−50	−38
passed	AgPat	44	48	−4	0
peeved	StimExp	10	102	−82	77
penalized	AgEvo	8	113	−87	−77
persecuted	AgEvo	9	110	−85	−22
petted	AgPat	6	114	−90	−30
picked up	AgPat	17	58	−55	−71
pitied	ExpStim	109	12	80	−83
placated	AgPat	10	99	−82	−7
plagued	StimExp	5	115	−92	58
played	AgPat	12	57	−65	43
played with	AgPat	17	94	−69	−13
pleased	StimExp	20	93	−65	83
praised	AgEvo	13	106	−78	−87
prized	ExpStim	54	58	−4	−74
prosecuted	AgEvo	11	102	−81	−44
protected	AgPat	11	103	−81	−47
provoked	AgPat	5	105	−91	70
punished	AgEvo	3	117	−95	−76
pursued	AgPat	26	63	−42	31
questioned	AgPat	16	99	−72	26
reassured	StimExp	6	112	−90	−62
rebuked	AgEvo	21	84	−60	−18
recompensed	AgEvo	13	80	−72	22
relaxed	StimExp	3	108	−95	19
relished	ExpStim	70	31	39	−47
remunerated	AgPat	16	78	−66	−6
repaid	AgPat	28	62	−38	63
repelled	StimExp	16	85	−68	67
reprimanded	AgEvo	8	109	−86	−50
reproached	AgEvo	13	100	−77	−12
reproved	AgEvo	18	90	−67	−14
repulsed	StimExp	23	96	−61	76
resented	ExpStim	95	9	83	−76
respected	ExpStim	78	25	51	−91
revered	ExpStim	79	33	41	−57
reviled	AgEvo	17	92	−69	−9
revitalized	StimExp	9	103	−84	3
revolted	StimExp	24	91	−58	66
rewarded	AgEvo	11	107	−81	−85
ridiculed	AgEvo	9	111	−85	−58
rushed to	AgPat	39	59	−20	−32
saluted	AgEvo	15	103	−75	−48
scared	StimExp	10	111	−83	74
scolded	AgEvo	6	113	−90	−69
scorned	AgEvo	9	100	−83	−49
shadowed	AgPat	69	49	17	58
shamed	AgPat	4	108	−93	12
shocked	StimExp	18	97	−69	56
shook	StimExp	13	109	−79	−47
sickened	StimExp	20	97	−66	67
slandered	AgEvo	9	98	−83	11
snubbed	AgEvo	10	101	−82	20
spanked	AgPat	4	117	−93	−72
spooked	StimExp	7	113	−88	62
staggered	StimExp	15	97	−73	64
stared at	AgPat	6	116	−90	−15
startled	StimExp	10	111	−83	35
stimulated	StimExp	5	98	−90	30
struck	AgPat	16	104	−73	−8
sued	AgEvo	22	84	−58	−77
supported	AgEvo	17	90	−68	−29
surprised	StimExp	13	103	−78	10
tailed	AgPat	42	77	−29	58
tantalized	StimExp	13	105	−78	48
telephoned	AgPat	12	49	−61	82
thanked	AgEvo	16	95	−71	−92
toasted	ExpStim	14	59	−62	−64
tolerated	ExpStim	48	24	33	4
took away	AgPat	15	69	−64	−50
tormented	StimExp	6	112	−90	45
tracked	AgPat	60	52	7	36
trailed	AgPat	40	75	−30	66
treasured	ExpStim	89	25	56	−76
troubled	StimExp	12	110	−80	68
trusted	ExpStim	90	15	71	−49
unnerved	StimExp	10	106	−83	70
unsettled	StimExp	15	106	−75	62
uplifted	StimExp	7	109	−88	−1
upset	StimExp	38	83	−37	66
valued	ExpStim	87	20	63	−98
venerated	ExpStim	60	49	10	−32
vexed	StimExp	12	99	−78	60
victimized	AgEvo	17	97	−70	15
vilified	AgEvo	14	80	−70	26
visited	AgPat	14	61	−63	−45
wanted	ExpStim	114	5	92	−26
warned	AgPat	12	110	−80	−17
wearied	StimExp	22	92	−61	65
welcomed	AgEvo	20	74	−57	−17
worried	StimExp	112	8	87	76
worried about	ExpStim	16	105	−74	−71
worshipped	ExpStim	61	50	10	−54
wounded	StimExp	23	86	−58	33
wowed	StimExp	14	104	−76	74
yearned for	ExpStim	103	16	73	13
yelled at	AgPat	6	111	−90	−57

## Footnotes

1 We note that the term “implicit consequence” is not as felicitous when applied to the person associated with the consequence, rather than the consequent action or state, as the term “implicit cause” used in a similar way. We will, however, adopt the convention of referring to this person as the implicit consequence.

2 This website was accessed in 2008, as indicated in the bibliography, but is no longer available. The names were extracted at that date for the causality study (Ferstl et al., 2011). The original causality norms paper appears to suggest that 305 pairs of common names were available. That is incorrect, and only 90 names of each sex were deemed common and ambiguous enough to be used.
